# Design of a Randomized Sham-Controlled Trial: Strengthening Positive Treatment Expectations Using a Communication Model for Maximized Antiemetic Effects of Acupuncture and Antiemetics During Emetogenic Neo-/Adjuvant Chemotherapy

**DOI:** 10.1177/15347354251361464

**Published:** 2025-08-22

**Authors:** Widgren Ylva, Fransson Per, Englund Erling, Efverman Anna

**Affiliations:** 1 University of Gävle, Sweden; 2 Sundsvall Hospital, Sweden; 3 Umeå University, Sweden; 4 Research and Development, Region of Västernorrland, Sweden

**Keywords:** communication, context, emesis, expectancy, nausea, placebo, vomiting

## Abstract

**Background:**

Chemotherapy-induced nausea and vomiting is a common problem in patients undergoing chemotherapy, influencing quality of life (QoL) and daily activities. This study will investigate whether therapists’ positive communication may strengthen positive treatment expectations and induce antiemetic effects during antiemetic treatment using standard care, sham acupuncture, or verum acupuncture, compared to neutral communication. It will also investigate whether a variety of patient, therapist and treatment components modify the treatment outcomes.

**Methods:**

This is a trial protocol for a randomized, sham-controlled, patient- and evaluator-blinded clinical trial. Patients (n = 198 patients according to a sample size calculation) with breast, colorectal, or bladder cancer undergoing neo-/adjuvant moderately to highly emetogenic chemotherapy are being randomized in 2 × 3 factorial design to the following conditions: 1. neutral or 2. positive communication style regarding expected treatment effects, during the antiemetic treatment types: A) standard care only (including antiemetics, no acupuncture), or, in addition to standard care, B) sham acupuncture with telescopic non-penetrating needles, or C) verum, penetrating acupuncture. The 2 communication styles 1 and 2 are carried out during the antiemetic treatments given by an intervention therapist immediately pre and post (20 minutes × 2) an intravenous chemotherapy session. Data are being collected at baseline on the day before the chemotherapy session, daily for 10 days, at a 10-day follow-up, and at a follow-up after completing the entire chemotherapy period, lasting about 6 months. Primary outcome is mean score Visual Analog Scale nausea grading for the first 5 days of the chemotherapy session period. Secondary outcomes are for example, vomiting, treatment expectations, QoL, daily and physical activity, and physiological measures. Treatment effect modifiers will be analyzed, for example, blinding success, treatment expectations, and previous nausea experiences.

**Conclusions:**

This trial will expand integrative cancer care’s understanding of the effects of communication for strengthening treatment expectations and thus alleviating chemotherapy-induced nausea and vomiting. If proven effective, the communication model of strengthening positive treatment expectations in patients with risk for nausea and vomiting can be implemented in routine clinical care as part of side-effect management for patients with cancer.

Trial registration: US National Institutes of Health, https://clinicaltrials.gov/study/NCT03232541?term=NCT03232541&rank=1), # NCT03232541.

## Introduction

Despite modern antiemetic treatment,^1-4^ nausea and vomiting are still considered one of the worst side effects of chemotherapy.^5^ These side effects often worsen patients’ quality of life (QoL),^6-9^ impair daily activities,^7,9,10^ and generate substantial healthcare costs,^11^ as well as financial burdens for patients, families, and society as a whole.^12^

Approximately 50 to 80 percent of patients experience nausea and/or vomiting during each chemotherapy cycle.^1,13^ These side effects usually occur within 1 to 5 days and can continue up to around 10 days.^1,2^ Although antiemetics play a crucial role in effective nausea and vomiting control,^14^ some patients receive inadequate treatment.^3,5,15^ Some patients even decline antiemetic prophylaxis.^16^ Without prophylactic medications, moderately emetogenic chemotherapy is associated with nausea and vomiting in 30% to 90% of patients. When using highly emetogenic chemotherapy, nearly all patients experience nausea and vomiting in the absence of adequate prophylaxis.^17^

Many patients request non-pharmacological treatments during cancer therapy: More than a fourth of 755 patients undergoing cancer therapy used complementary and alternative medicine (CAM) after receiving their cancer diagnosis.^18^ Acupuncture is the most frequently offered complementary treatment within established cancer care in Western countries,^19,20^ and on the top 3 worldwide.^21^ Patients report great interest in receiving acupuncture for side effects during their cancer therapy^22^ and cancer care personnel highly believe acupuncture to be effective for various cancer-therapy induced side-effects.^23^ Guidelines^24,25^ recommend acupuncture to the target population. Despite great interest in acupuncture among patients and personnel^22,23^ and widespread implementation across oncology clinics,^19-21^ there is still limited scientific evidence^25,26^ concerning the method’s specific effects. Few studies have been designed to distinguish *specific effects*, resulting from skin penetration and needle stimulation, from effects of *nonspecific* treatment components, such as treatment expectations and communication during treatment,^27^ by adopting a credible sham-controlled design. These studies have presented contradictory results regarding nausea and vomiting outcomes: both positive results^28^ and negative results have been observed.^29^ Other recent studies have unfortunately not applied any control treatment,^30,31^ or used verum acupuncture as a control treatment, the only difference being that the needles were inserted at non-traditional acupuncture points.^32^ Hypotheses, yet unconfirmed by scientific studies,^33^ have been proposed stating that acupuncture may have specific effects against nausea and vomiting by inhibiting impulses from the vagus nerve to the vomiting center or by increasing patients’ cortisol concentration.^34^ Elevated cortisol concentration may plausibly prevent nausea, potentially through the same mechanisms that corticosteroids (converted to cortisol in the body) do.^1^

The underlying mechanisms of chemotherapy-induced nausea involve both biological and psychosocial mechanisms.^35^ The risk of nausea is elevated in anxious^2,4^ or hopeless patients^36^ and in patients who believe they are at high risk of experiencing nausea,^36-38^ for example, based on previous nausea experiences.^36^ Treatment expectation^37^ has the potential to modify effects of integrative cancer therapies, acupuncture included. However, acupuncture studies often fail to measure treatment expectations.^39^ Responders to acupuncture described themselves to have a more trustful relationship to their therapist than did non-responders.^40^ Patients described expectations to be important for shaping new experiences of nausea during chemotherapy.^9^ This gives a hypothetical basis for potential antiemetic effects of adopting the non-specific treatment component communication to strengthen positive expectations concerning the effects of a treatment,^41,42^ that is, antiemetic treatment. Non-clinical experimental studies have observed expectancy-related effects on nausea, also in stressful conditions.^43^ However, how patients’ expectations and symptom experience influence each other over time is still unknown, raising a question of whether personnel can strengthen patients’ positive expectations through their communication and thus prevent or alleviate symptoms in standard care settings.^39,41,44-46^ A previous study observed a dramatic effect of sham acupuncture in a non-cancer population with bowel health symptoms, especially when given by therapists employing empathic, confidence-instilling communication during treatments.^47^ Inspired by that study,^47^ we have developed a communication model, involving verbal communication with corresponding non-verbal communication^48,49,50^ intended to strengthen positive treatment expectations. We evaluated the model’s validity in non-cancer individuals. The individuals who had the best effect, were those who, owing to the therapist’s communication, managed to increase their treatment expectations the most.^51^

Previous findings^27,47,51^ have shown that acupuncture treatment and non-specific components of the caring situation have great potential to produce valuable effects; these findings need to be evaluated in a scientific study that employs good methodology. Converging observations from previous studies^39,41,44-46^ have suggested that non-specific treatment components can be explored and used systematically, and that this may benefit patients significantly. There is a lack of knowledge regarding if strengthening treatment expectations is effective in clinical contexts.^39,44-46^ More knowledge is also needed about health economic benefits of antiemetic acupuncture^30^ and interventions based on non-specific treatment components.^52^

In the present protocol, we describe a randomized, sham-controlled, patient- and evaluator-blinded trial to examine the efficacy of a communication model intended to strengthen positive treatment expectations among patients during antiemetic treatment for chemotherapy-induced nausea and vomiting. The twofold objective is to study whether nausea and vomiting, QoL, and daily and physical activities differ between patients with cancer who, during a chemotherapy, receive:

1. Neutral or 2. positive communication about the expected antiemetic effects while undergoing:
A. Standard care with antiemetics only, or, added to standard care, B. sham acupuncture, or C, verum acupuncture.

The main study hypothesis is that positive communication plus acupuncture (penetrating verum or non-penetrating sham acupuncture) is more effective than neutral communication plus standard care for nausea, calculated as the mean score for the first 5 days of the studied chemotherapy session period. We hypothesize that treatment expectation is a mediator of the effects.

## Methods

### Design

**Figure 1.**
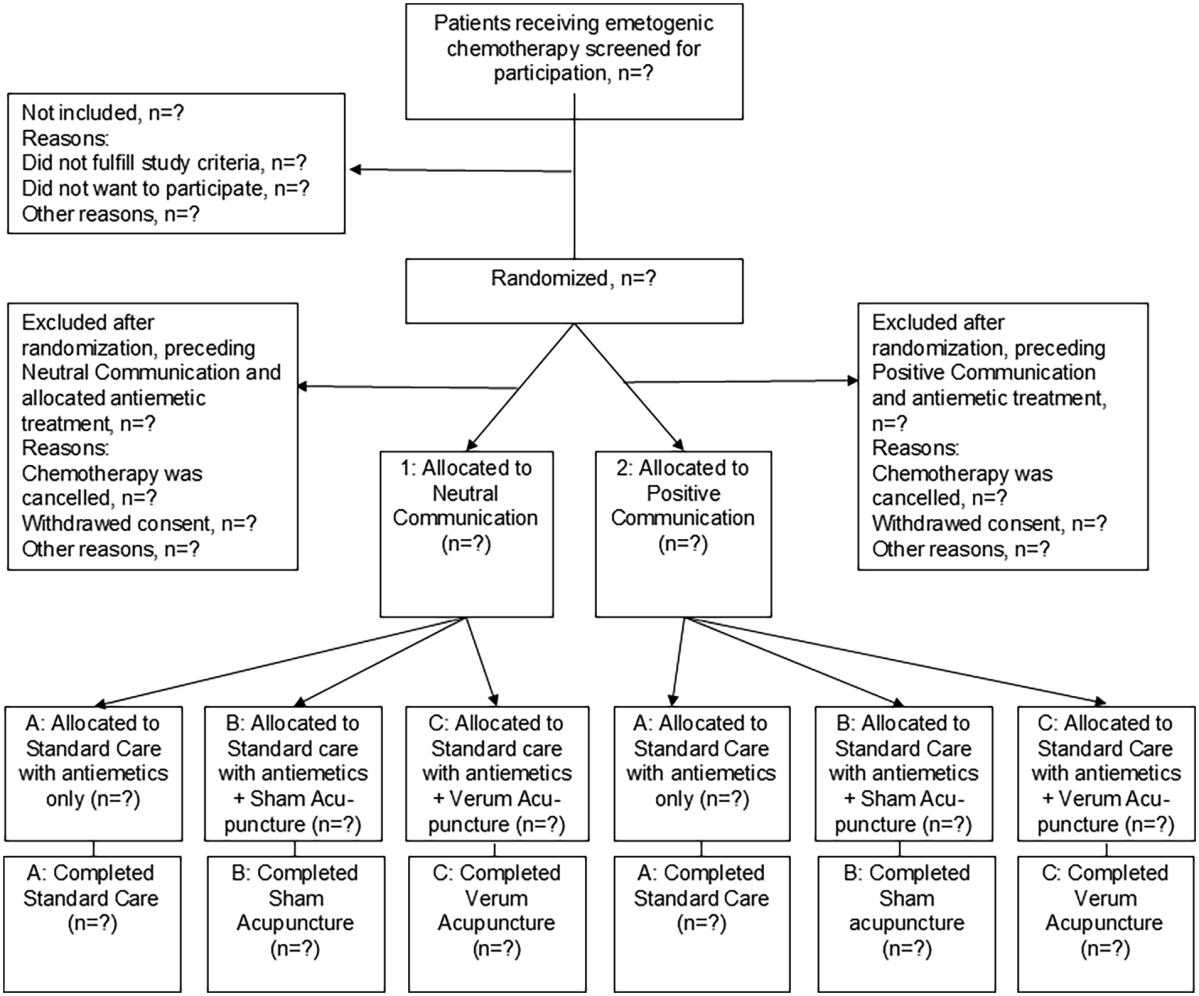
Flow chart of patient inclusion and allocation groups. The intended distribution is 1:1, meaning approximately equal numbers will be allocated to each arm.

The study uses a randomized sham-controlled design. The design is a 2 × 3 factorial design^53^ where patients are randomized to the communication styles 1. neutral communication, or 2. positive communication, given when undergoing 1 of 3 antiemetic treatments (Figure 1). All patients receive standard care; patients randomized to antiemetic treatment type B (sham acupuncture), or C (verum acupuncture) receive the acupuncture therapy in addition to A (standard care). The structure of the intervention follows the Template for Intervention Description and Replication (TIDieR) checklist^54^ and the Standard Protocol Items: Recommendations for Interventional Trials (SPIRIT).^55^

The study was approved by the Regional Ethical Committee (Umeå 2016/362-31, and Umeå 2017/488-32) and trial registration was conducted preceding the inclusion of the first participant.

### Sample Size

Participants will complete the study procedure during a single chemotherapy session, specifically, the next scheduled session following inclusion. The primary outcome is the mean intensity of nausea for the first 5 days, that is, the day of the intravenous chemotherapy session and the 4 following days of the studied chemotherapy session period. We performed an a priori sample size calculation, ensuring the study to be sufficiently powered. Based on previous research,^15^ we aimed to detect a clinically important difference of 10 millimeters (mm) using a 100 mm Visual Analog Scale (VAS) nausea measure between the positive and neutral communication group. Assuming a 2-sided alpha of .05 and statistical power of 80%, we estimated that 198 participants (99 per communication style group) would be required. The data analysis will be conducted according to intention to treat. To allow for a potential dropout rate of 20% between inclusion and the study procedure, the study will recruit up to 240 patients.

### Recruitment of Patients

Patients are consecutively being recruited from 3 hospitals, located in northern and central of Sweden. Inclusion criteria are breast, colorectal, or bladder cancer, age ≥ 18 years, scheduled for adjuvant or neo-adjuvant intravenous chemotherapy of moderate or high emetogenicity^17^ (Table 1), and physical, mental and linguistic capacity to give informed consent and participate in the study procedure, that is, understand information in the language of the study, that is, Swedish. Exclusion criteria include the presence of persistent nausea or ongoing antiemetic treatment before the very first chemotherapy session—specifically, if symptoms persist into the day before initiating chemotherapy—as well as a diagnosis of hemophilia. Additionally, any prior participation in the study (such as during a recurrence or following relocation from another city) will exclude patients from enrollment.

**Table 1.** The Chemotherapy Agents.

Chemotherapy agents	Highly emetogenic chemotherapy agent, HEC	Moderately emetogenic chemotherapy agent, MEC	Low emetogenic chemotherapy agent, MEC
Cisplatin	X		
Carboplatin		X	
Irinotecan		X	
Cyclophosphamide <1500 mg/m^2^		X	
Doxorubicin		X	
Epirubicin		X	
Oxaliplatin		X	
Mitomycin			X
Paclitaxel			X
Docetaxel^a^		X	
5-Fluorouracil			X
Gemcitabine			X

Some patients receive LEC combined with MEC or another LEC. When 2 or more agents are combined the emetogenic potential essentially becomes 1 grade higher than that of the most emetogenic agent in the combination. Those patients thus receive highly or moderately emetogenic chemotherapy and are eligible for this study.

a Considered as moderately emetogenic according to the local guideline implemented at the studied clinics like in previous versions of international guidelines, applied when starting the data collection. The recently updated guideline^17^ considers Docetaxel to be of low emetogenicity.

Oncology nurses at the oncology departments are consecutively identifying patients scheduled for chemotherapy, asking if the patients are interested in receiving information about the study, and if they are, the nurses send study information by post. Next, the study coordinator of each department contacts the patients, providing the study information verbally and giving opportunities to ask questions. The written and verbal information states that patients will be randomized to receive antiemetics according to standard care routines or standard care combined with 1 of 2 types of acupuncture needling procedures: one that penetrates the skin and one where the needles are placed against the skin. Neither of the acupuncture needling procedures is described as a sham treatment. Further, the information states that the treatment types will be given using 2 types of “treatment procedures” performed by the intervention therapist, without any further specification, the goal being to prevent patients from knowing too much about the exact contents of the communication styles. Patients who agree to participate in the trial give their written informed consent.

Participants are scheduled to complete the study procedure during the chemotherapy session immediately following inclusion. However, certain exceptions to this plan may occur. The combination of chemotherapy agents varies according to each patient’s individual cancer treatment plan. If the next scheduled session involves only low-emetogenic agents (ie, no moderate-to-high emetogenicity), the study procedure will instead take place during the subsequent session. Practical considerations—such as treatment being administered at a different hospital or the intervention therapist being unavailable—may also necessitate postponing the study procedure to a later session. In these cases, participants will be involved in the study procedure during the next appropriate chemotherapy session. We will document the total number of chemotherapy sessions administered prior to the study session and record the emetogenicity levels of the agents used (see chapter Data collection, on medical record clinical data).

### Randomization and Blinding

**Figure 2.**
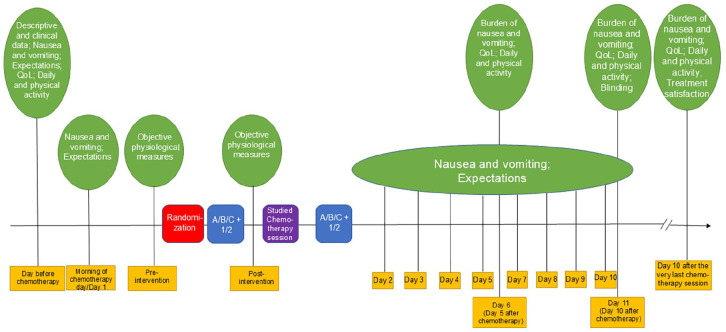
Timeline of data collection. T1, Time-point 1, T2, Time-point 2. Antiemetic treatment types: A, standard care, no acupuncture; B, sham acupuncture; C; verum acupuncture. Communication styles: 1, neutral communication style; 2, positive communication style. Objective physiological measures: heart rate, blood pressure, salivary cortisol. Repeatedly during the study period, also co-occurring symptoms, and co-interventions such as medications and self-care are self-registered.

Using a computerized randomization table, a statistician randomizes the patients, stratified for hospital, to 1 of the 2 communication styles (1 or 2) conducted during 3 treatment types (Type A, B, or C; Figure 1) without applying any block randomization. After the intervention therapist initially conducts the physiological measures described below, he/she receives the randomization result from the randomization table and begins the study procedure in line with the randomization result (Figure 2), according to a standardized treatment protocol. The patients and all medical and healthcare personnel, other than the intervention therapist delivering the standardized treatment protocol procedures, are blinded to the acupuncture and communication allocation (see description of treatment types below). The evaluator is blinded to allocation until after analysis of the primary outcome. This is possible because all patients and allocation groups are coded; the evaluator does not know which code means which allocation group until thereafter.

### Study Procedure

The intervention therapist conducts the treatment procedure twice within the studied chemotherapy session. The first time (T1), illustrated with 1/2 + A/B/C in Figure 2, is conducted for 20 minutes (irrespective of verum or sham acupuncture is given) immediately pre intravenous chemotherapy. The second time (T2), also illustrated with 1/2 + A/B/C in Figure 2, is conducted for 20 minutes (irrespective of verum or sham acupuncture is given) immediately post intravenous chemotherapy. Twenty to 30 minutes is a common duration for a single acupuncture session in clinical practice.^23^ If the patient’s chemotherapy session lasts for 2 consecutive days, the intervention takes place on day 1. The whole study procedure lasts from the day before intervention day until day 10 after the very last chemotherapy session. Figure 2 presents the study procedure timeline.

### The Intervention Therapists Delivering the Communication Model and the Acupuncture Types

The therapists delivering the communication styles and the antiemetic acupuncture types (the intervention therapists) are registered physiotherapists with professional experience and education in acupuncture. Before beginning as an intervention therapist for the present study, they are introduced to the standardized treatment protocol for a full working day (about 8 hours) by the project manager (the last author). They then continue to practice until they feel comfortable and experienced in implementing the standardized study protocol. Prior to the study, some of the intervention therapists have also practiced the communication model with 30 study participants each, during the preceding procedure validating the communication model.^51^ The intervention therapists respond to a study-specific questionnaire regarding years in the profession and their own experience of and belief in acupuncture effects prior to joining the study. The treatment expectancy measure,^56^ ask: “Do you believe that the treatment you are going to give is effective against nausea and vomiting?” rated on a 100 mm VAS anchored with 0 (“I don’t believe at all that it’s effective”) and 100 (“I completely believe that it’s effective”).

### The Communication Model Involving 2 Communication Styles

To manipulate the non-specific component treatment expectations during treatments,^57^ the intervention therapist applies a previously validated communication model,^51^ inspired by Kaptchuk et al,^47^ covering 2 verbal communication styles, where the non-verbal communication (facial expressions and body language)^48,49^ is in line with the verbal communication.

1. The neutral communication style: During treatment Type B-C at T1 and T2, the intervention therapist communicates at least 3 out of several neutral statements such as: “Many acupuncture studies have shown different results regarding whether or not acupuncture treatment prevents and relieves nausea,” “We don’t really know if acupuncture is a good method for producing antiemetic effects; thus we need to perform this study,” “We cannot know whether you will experience antiemetic effects of the acupuncture treatment; I have treated persons who felt differently about it.” During treatment Type A, the neutral communication is to be as similar to standard care as possible. The intervention therapist just quickly comes around, explains the data collection procedure, but does not talk explicitly about expected antiemetic effects.
2. The positive communication style: The intervention therapist communicates at least 3 of several positive statements during treatment Type B-C such as: “Many acupuncture studies have shown that acupuncture is effective compared to medication alone,” “Patients receiving acupuncture often feel that the treatment prevents and relieves nausea,” and “I have had positive effects preventing nausea using this treatment before.” During treatment Type A, the intervention therapist communicates things such as: “Many studies show that antiemetics are highly effective in preventing and relieving nausea,” “Many patients who receive the antiemetic medication you are receiving feel that the medication prevents and relieves nausea,” and “I have met many patients who have had good effects from their antiemetics.”

The positive or neutral communication is to permeate the entire intervention. The intervention therapists are instructed to find a personal, natural way to deliver the content of the statements described above, rather than repeating them verbatim.

### The Antiemetic Treatment Involving 3 Treatment Types

When undergoing A, B, or C, the patient (not undressed) is in the position for receiving intravenous chemotherapy, that is, sitting relaxed or lying.

A) The standard care group receives no acupuncture. Patients receive only antiemetics, according to the oncology departments’ standard care routines (at doses according to the Swedish Medicine Information Engine; http://www.fass.se) based on international guidelines,^1-4^ adapted to individual patients^17^ by their oncologists.
B) The intervention therapist administers *sham acupuncture* for 20 minutes with the telescopic non-penetrating Park et al’s sham needle (blunt needles diameter 0.25 × length 45 mm)^58^ bilaterally to a non-acupuncture point 1 body-inch radial to the PC6 point, which is located 4 body-inches proximal to the wrist. Park’s sham needle credibly blinds patients.^59^ It looks identical to a real needle but glides upward into its handle, giving the illusion of penetration.^58^ A marking tube, identical for both acupuncture types, holds the sham needle in place (the marking tube is used in both allocation groups). The intervention therapist manipulates the sham needle a few seconds 3 times per session until the needle touches the skin, but no “needle sensation” occurs, and then lifts the needles up from the skin. Except for when placing and manipulating the needle, the sham needle thus puts no pressure on the skin, like the procedure used in previous studies.^27,37,51^
C) The intervention therapist administers western medicine *verum acupuncture* (sharp needles diameter 0.25 × length 40 mm) for 20 minutes bilaterally to the acupuncture point pericardium six (PC6). PC6 is located between the tendons of palmaris longus and flexor carpii radialis at 2 body-inches (1 body-inch approximately 1.5 cm) proximal to the wrist at 0.5 body-inch depth. The therapist manually manipulates the needles 3 times per treatment T1 and T2 by rotating, thrusting, or lifting the needles. When patients report a sense of numbness or soreness and the therapist observes a minimal muscular contraction around the needle, that is, the “needle sensation.”^60^

### Data Collection

**Figure 3.**
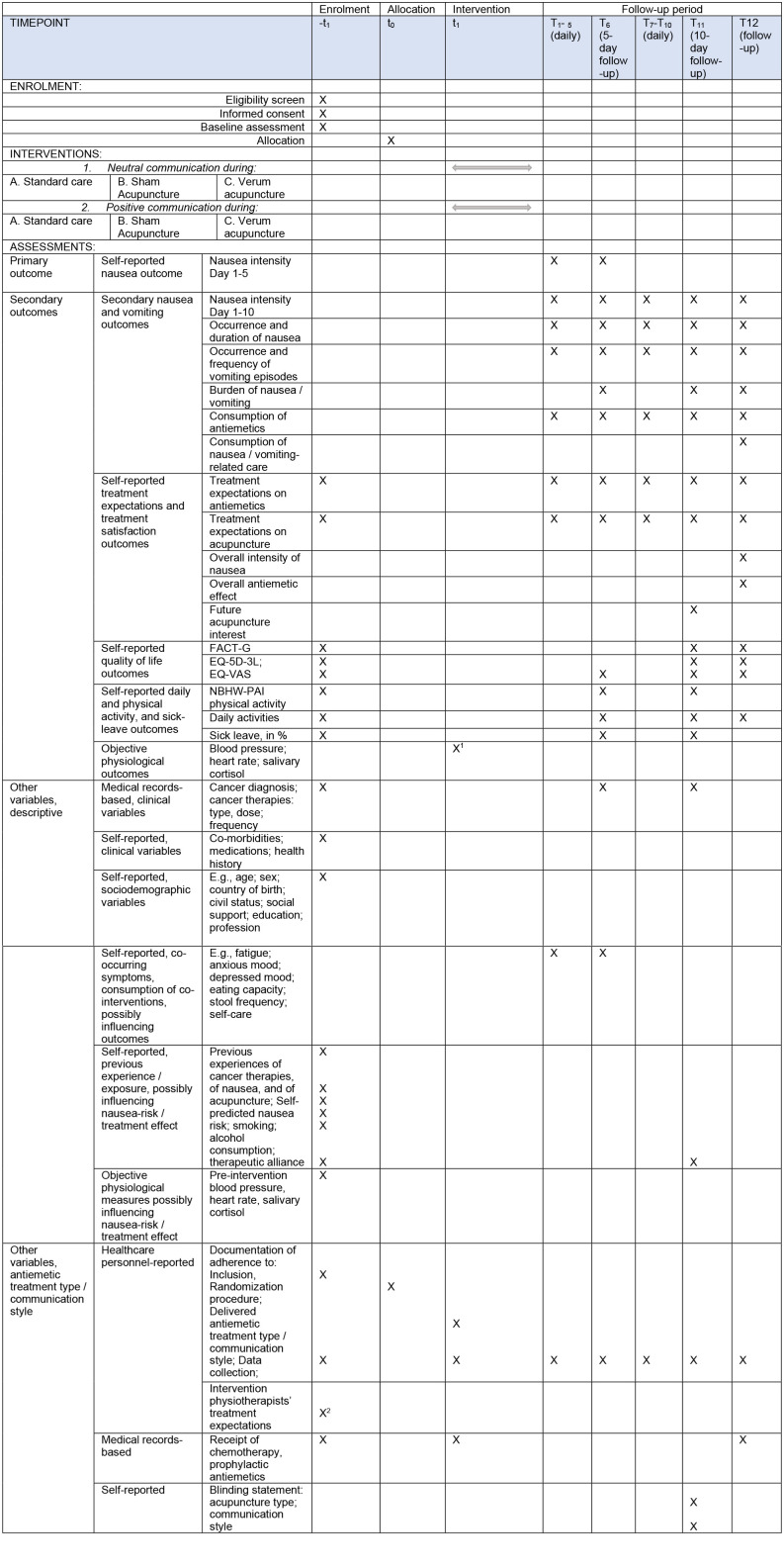
Schedule of enrollment, intervention and assessment (SPIRIT). ^1^Measured immediately post intervention. ^2^Therapist data were only collected once, preceding study involvement. FACT-G: Functional Assessment of Cancer Therapy-General; QoL: quality of life; EQ-5D-3L: Euroqol 5 dimensions, 3 levels; EQ-VAS: Euroqol Visual Analog Scale; NBHW-PAI: National Board of Health and Welfare’s Physical Activity Indicator Questions.

Data are being collected at multiple time points (see Figure 2): first, at baseline—the day before the designated chemotherapy session in which the patient participates in the study; then daily for 10 consecutive days, with explicit follow-ups on day 5 and day 10; and finally, 10 days after completion of the patient’s entire chemotherapy regimen (which typically lasts about 6 months according to the individual care plan). The SPIRIT^54^ Figure 3 gives an overview of the data collection.

Independent of patients’ cancer care, the study coordinator at each department sends the baseline questionnaire and the daily nausea and vomiting measure to patients by post or e-mail, according to the preferences patients expressed during the telephone call when study information was provided. The intervention therapists give patients the 10-day follow-up questionnaire and the completion questionnaire directly after conclusion of the study intervention, clearly stating that “these questionnaires come from the evaluator, and I will not be aware of your answers.” Patients may answer the different questionnaires using either “pen and paper” or the study’s digital web questionnaires, according to their own preferences. The study information ensures patients that their responses (marked by an identification code instead of any personal information) will not be managed by the cancer care personnel, only by the independent study evaluator.

About 1 to 2 weeks after an expected response, the study coordinator sends a gentle reminder through the post to those who have not yet responded, and after responding to the completion questionnaire, patients receive a letter, sent via post, expressing our gratitude for their participation. Figure 3 presents the measurements described below.

Medical record clinical data: Research nurses of the oncology departments collect data from patients’ medical records regarding cancer diagnosis, disease-specific variables, types, doses and frequency of chemotherapy, and if the patient was chemotherapy-naïve or not when participating in the study. Data on the types and doses of prophylactic antiemetics given to the patients are also collected, for the entire chemotherapy period.

Self-reported descriptive data: On a baseline questionnaire, patients supply sociodemographic data (eg, sex, age, level of education, profession), clinical data (eg, comorbidities, disease-specific variables, medications), data on previous experience of and belief in acupuncture effects,^22^ data on previous nausea experiences in their lives and specifically when undergoing cancer therapy, self-predicted risk for nausea,^36^ and data regarding therapeutic alliance, for example, to which extent the patient trusts the therapist to be able to help him/her.^61^

Data on nausea and vomiting: Patients complete a measure of nausea and vomiting^7^ daily for 10 days, answering questions about the primary outcome (nausea intensity) and the secondary outcomes (nausea occurrence, duration of nausea, vomiting episodes, and consumption of antiemetics). This measure asks the following questions with reference to the past 24 hours: “Have you experienced nausea?” (“No” / “Yes”); “How intense was the nausea?” A 100 mm VAS with verbal anchors “No nausea” and “Worst possible nausea” is used. The accumulated duration (in minutes) of nausea within the past 24 hours is registered. Patients also respond to the questions: “Have you vomited?” (“No”/“Yes”), “How many episodes?” (number of episodes is given), and “Have you consumed rescue antiemetics?” (“No”/“Yes”). They are asked to: “Please detail agent, dose and frequency of each antiemetic agent used.” Also, other kinds of nausea and vomiting-related care consumption is self-registered. Burden is asked for and registered on 2 separate 100-mm VAS: “Did the nausea negatively affect your daily life?.” The same question is posed regarding vomiting, both anchored with 0 (“Not affected at all”) and “100” (“Affected in the worst possible way”; Figure 2).

Expectations data: Patients rate their treatment expectations daily for 10 days, using a valid expectancy measure,^56^ “Do you believe that the treatment you are going to receive is effective for nausea and vomiting?” This is measured using a 100 mm VAS anchored with 0 (“I don’t believe at all that it’s effective”) and 100 (“I completely believe that it’s effective”). Post-treatment, the question is written with the modified verb tenses “received” and “was.”

Treatment satisfaction data: On day 10 after the intervention, patients rate their interest in receiving future acupuncture treatment by answering the questions^37^ “If you should need similar chemotherapy in the future, would you be interested in receiving antiemetic acupuncture treatment?” (“Yes, completely interested,” “Yes, very interested,” “Yes, moderately interested,” “Yes, a little interested,” “No, not interested”), and “If you should experience other problems in the future that could be treated with acupuncture, would you be interested in receiving acupuncture treatment?” (“Yes, completely interested,” “Yes, very interested,” “Yes, moderately interested,” “Yes, a little interested,” “No, not interested”). After completion of the entire chemotherapy period, patients rate the self-perceived overall nausea intensity, and the burden of it, for the entire chemotherapy period,^7^ as well as the overall perceived effect of the antiemetic treatment,^37^ using 3 different 100 mm VAS anchored with 0 and 100 (labeled “No nausea” and “Worst possible nausea”; “No burden” and “Worst possible burden”; and “Not effective at all” and “Completely effective,” respectively).

QoL data: Patients repeatedly (Figure 2) respond to the Swedish version^62^ of Functional Assessment of Cancer Therapy—General (FACT-G).^63^ The 27-item FACT-G covers 4 domains on health-related QoL: physical wellbeing (7 items), social wellbeing (7 items), emotional wellbeing (6 items), and functional wellbeing (7 items), graded on a numerical rating scale from 0, “completely disagree” to 4, “completely agree.” Higher scores on the domains and the total score indicate better QoL. Patients also grade their health-related QoL on the Swedish version^64^ of the Euroqol-5 Dimensions-3 Levels (mobility, self-care, usual activities, pain/discomfort, anxiety/depression), and Euroqol-VAS, a 100 mm vertical scale anchored with 100, “best imaginable health state” and 0, “worst imaginable health state.”^65^ Further, responses are given to a single-item question regarding global QoL.^66^

Daily and physical activity data: Daily activities (often mentioned as “everyday activity,” or “activities of daily living”) the preceding week are assessed using 7-graded statements ranging from “I managed all my daily activities” to “I did not manage any of my daily activities.”^67^ Physical activity performance is assessed using widely used self-report questions^68^: “During a regular week, how much time do you spend exercising at a level that makes you short of breath, for example running, fitness class, or ball games?” (ie, vigorous activity), and “During the past week, how much time are you physically active in ways that are not exercise, for example walk, bicycling, or gardening?” (ie, moderately intensive activity). Patients answer the questions by adding together all activities lasting at least 10 minutes and choosing between categories, for example, “31-60 minutes.” At baseline, they register vigorous and moderate physical activity during a regular week, using the same questions regarding the current week.^68^ They report their work activity by self-reporting the percentage of sickness absence (0%, 25%, 50%, 75%, or 100% sick leave), which has good agreement with register-based data.^69^ Using a single-item question, potential fear of being active (kinesiophobia)^70^ is registered.

Objective physiological data: The intervention therapist measures patients’ heart rate (Beats Per Minute, BPM) and blood pressure (mm of mercury, mm/Hg) twice, using a digital upper arm blood pressure monitor twice (Figure 2). Patients place their left arm, which is relaxed, on the surface (with their palm up), and the intervention therapist positions the monitor approximately 2 cm above the elbow joint.

Patients provide saliva samples twice (Figure 2) for measuring cortisol concentrations, according to previously used standardized routines.^71^ They perform the first sampling procedure after resting during an introductory conversation and pre-chemotherapy preparations with the intervention therapist (approximately 10-15 minutes) immediately preceding their treatment A, B, or C, combined with communication style 1 or 2. They wet a cotton swab with saliva by moving the swab around in their mouth for 1 to 2 minutes, without eating, drinking, chewing gum, or using tobacco during this procedure. They then place the swab in a Salivette^®^ tube (Sarstedt, Nümbrecht, Germany). The patients perform the second sampling procedure according to the same standardized routine immediately after their treatment A, B, or C, combined with communication style 1 or 2.

The analyzer at the laboratory of each hospital at the study sites analyzes the samples using an enzyme immunoassay kit (Salimetrics LLC, USA). Regarding the precision of the measurements, the total coefficient of variation is 13.3% at 3.4 nmol/L and 6.3% at 29.4 nmol/L. The detection limit is 0.3 nmol/L. If the analyses cannot be done consecutively, the laboratory may store the samples at −20°C for up to 9 months.^71^ In the event of storage, the samples will be thawed at room temperature and centrifuged at 1500*g* before assay.

Treatment adherence, complications, and blinding data: At every treatment session, the intervention therapists register their adherence to the study protocol (eg, treated acupuncture points, number of needle stimulations, delivered verbal phrases). The intervention therapists register potential negative side-effects or complications occurring during the treatment sessions.

At the 10-day follow-up measure, patients respond in a binary fashion to a blinding measure^59^: “Do you think you have been treated with needles that have penetrated your skin, or do you think the needles have only been placed against the surface of your skin?” They also grade how certain they feel on the accuracy of their response: “Not sure at all, just guessed,” “fairly sure,” “entirely sure.” If patients have received standard care only, they choose the additional alternative “Not applicable, I did not receive any acupuncture treatment.” They respond to the question^51^: “During your chemotherapy session, you met an intervention therapist. Do you think you have received treatment from a therapist who emphasized the expected positive effects or one who did not?” This is measured using a 100 mm VAS anchored with 0, “Communicated very negative” and 100, “Communicated completely positive.” Patients in treatment type B-C grade their self-perceived needle-induced pain (“No/little/moderate/very painful”) and register potential side-effects of the acupuncture treatment.

Co-occurring symptoms, consumption of co-interventions: Patients repeatedly grade experience of co-occurring symptoms/problems, fatigue,^72^ sleeping quality,^73^ anxious and depressed mood,^74^ relaxation,^51^ pain and unpleasant symptoms (ie, neuropathy symptoms),^75^ wellbeing,^37^ eating capacity,^76^ and stool frequency.^57^ Since both symptoms and treatments may influence nausea and vomiting, QoL, and daily or physical activity levels, we include study-specific questions to determine whether participants are taking medications or other co-interventions beyond antiemetics and to describe their potential self-care strategies.

### Data Analysis Plan

The intention-to-treat principle will be applied when analyzing the study data, that is, all patients will be analyzed as randomized, regardless of whether they complete the study procedure as planned. The primary parameters of interests are the 2 factorial effects (2 main effect, ie, 1. communication style, and 2. treatment type, and one interaction, ie, treatment expectations) during the first 5 days of the studied chemotherapy session period. The evaluator will use a generalized linear mixed effects model (GLMM) to determine if the independent variables communication style and treatment type affect the dependent variable nausea. We take the stratified randomization into account (adjusting for random effects of department on the outcomes) and include baseline characteristics in the analysis model to increase the precision of the estimates.^77^ In secondary GLMMs, we will study the effects of the variety of independent variables on vomiting, QoL, and daily and physical activity. Patients who cease to participate (dropouts) and missing data will be handled according to routines as follows. In the main analysis, missing data will be handled using multiple imputations by chained equation.^78^ We will specify the multiple imputation model (the choice of auxiliary variables) after comparing patient characteristics between those with complete and those with incomplete data. Under the assumption that data are missing at random, an analysis based on multiple imputation conforms with the intention to treat. We will then also estimate the factorial effects using complete data only. We will conduct a sensitivity analysis by comparing estimates regarding the 2 main effects described above with estimates based on complete data only, in other words, a pattern-mixture model and tipping point analyses. Statistical significance will be defined as a 2-tailed *P* < .05, and the analyses will be performed in IBM SPSS statistics (IBM Corp, Armonk NY, USA).

In line with previous recommendations regarding cost-effectiveness analysis alongside clinical trials, we will specify the methods for cost effectiveness analysis after evaluating these outcomes. If the present evaluation shows significant health benefits on the primary outcome measure, or in at least on 1 secondary outcome, a cost-effectiveness analysis of our intervention will be conducted using a model-based approach.^79^

External study monitoring will be conducted according to the guideline for good clinical practice adapted to non-medical studies. Independent observations will be conducted regarding the intervention physiotherapists’ compliance to the standardized treatment protocol during 1/2 + A/B/C, and the validity results will be presented.

## Discussion

The present study aims to determine whether therapists’ positive communication about antiemetic effects may strengthening treatment expectations when undergoing antiemetic treatment using standard care, sham acupuncture or verum acupuncture and thus affects nausea and vomiting, QoL, and capacity in daily and physical activity. Additionally, the study investigates the cost-effectiveness of this kind of communication and treatment procedure.

Systematic reviews have stressed the potential for benefits from antiemetic treatment procedures, which include acupuncture^24,25^ and caring procedures involving communication intended to reinforce patients’ hope and positive treatment expectations.^42,49^ Several researchers have pointed out the lack of knowledge regarding whether findings from non-clinical experimental studies on the effects of information and communication can also be seen in clinical settings with patients.^39,44-46^ Psychosocial interventions have the potential to induce clinically relevant effects on chemotherapy-induced nausea and vomiting,^80^ for example, information on how to manage symptoms during chemotherapy significantly improved patients’ QoL.^81^ A review showed that a positive outlook and uplifting spirit on the part of healthcare personnel gave patients hope and inspiration.^83^ However, we do not yet know whether the characteristics of the communication, positive or neutral, play any significant role in the antiemetic effects experienced by patients undergoing emetogenic chemotherapy.

The primary outcome of the present study is nausea, as it is one of the most burdensome side effects among patients undergoing chemotherapy.^5,15^ Based on patient-experiences supporting the beneficial effects of positive treatment expectations and communication during chemotherapy,^9^ the next natural step will be to present the current large-scale, well-designed study, addressing what constitutes optimal communication for obtaining effects on nausea and vomiting and for improving QoL and capacity in daily and physical activities among patients undergoing chemotherapy. The patients participate in the study during one chemotherapy session, and thus the primary outcome is based on data collected within that session. To intervene with the patients and thus collect data during the entire chemotherapy period for about 6 months was valued to be unethical. We want to minimize the burden of the study on this rather frail patient group until we become aware of the efficacy of the treatment types and the communication styles. If positive communication added to antiemetic treatment is effective, this integrative cancer therapy intervention may be implemented during the entire chemotherapy period. The underlying mechanisms of nausea and vomiting are complex and involve a wide range of biopsychosocial mechanisms.^35^ To identify potential *psychological* mechanisms mediating beneficial effects of antiemetic acupuncture, treatment expectations will be analyzed. To identify potential *biological* mechanisms underlying beneficial effects of antiemetic acupuncture, the stress indicators blood pressure, heart rate and cortisol concentration in saliva will be analyzed. The data collection procedures thoroughly collect data on previous nausea experiences, since exposure to emetogenic situations and to previous nausea increases the risk for developing nausea.^2,4,36^

The research team involving and surrounding the authors includes experts in integrative medicine, nursing, physiotherapy, psychology and behavioral medicine, oncology, cell biology, and health economy, thus enabling a broad-based approach to gain new knowledge. The study will contribute knowledge that can be used when treating patients in clinical practice by evaluating the role of healthcare personnel’s communication and patients’ treatment expectations, as well as the use of different antiemetic strategies, that is, using antiemetics alone or antiemetics plus acupuncture. Evaluating the effects of communication and antiemetic therapy as well as identifying moderating and mediating variables on our outcome—nausea—may be expected to be beneficial on at least 3 levels. Individual gains of reduced nausea may include improved self-perceived QoL,^6-9^ facilitated daily and physical activity,^7,10^ and perhaps a reduced need for rescue antiemetics.^76^ This in turn may result in lower burden on the healthcare system and reduced societal costs,^11,12^ thus positively affecting public health. Health economic evaluations of integrative interventions regarding effects of non-specific treatment components, sometimes cited as “placebo effects,” are scarce.^52^

Important aspects of the study plan are the randomized controlled design, and the accurate collection of data needed to be able to control for potential confounding factors. Although the main aim of the present effectiveness study is to compare different communication styles and to compare verum acupuncture with sham acupuncture, one important methodological choice was also to include a third treatment type group, that is, the standard care group. In line with recommendations for research on contextual effects,^83^ this allows us to compare how much additional nausea-reducing effect positive communication and acupuncture may have, if any, compared to the standard care that all patients receive in routine clinical practice. Given the strong evidence showing that pharmacological antiemetic therapy is beneficial for persons undergoing emetogenic chemotherapy,^1-4,14^ we considered it unethical to randomize persons to an untreated control group that would not be offered any antiemetic therapy at all. Accordingly, all patients receive standard care, and the verum and sham acupuncture and communication styles are conducted in addition to standard care. Patients randomized to standard care combined with neutral communication receive treatment that is comparable to the treatment given in clinical standard care. To succeed with the challenge of blinding patients, the study uses the credible^59^ telescopic Park et al’s sham device^58^ in the sham acupuncture group. Patients, acupuncture-experienced patients included, could not distinguish if they received verum acupuncture to PC6 or sham acupuncture, using that device.^59^ The chosen sham point is a non-acupuncture point, to avoid sensory stimulation of PC6. A recent complex network analysis valued the effect of acupuncture for chemotherapy-induced nausea and vomiting based on 489 acupuncture trials. PC6 and ST36 (at the knee) were the most frequently used single acupuncture points, of which PC6 was the most frequently used for specifically *nausea*,^84^ being the primary outcome of the currently described study. In routine clinical care, several patients often receive chemotherapy in the same room. Of the mentioned acupuncture points, PC6 is the only point that can be treated without undressing. Further, verum acupuncture at PC6 selectively activated neural responses of the insula, hypothalamus, and cerebellum compared to other acupuncture points.^85^ Verum acupuncture at PC6 was seen to selectively activate midbrain sensory areas, in comparison with sham acupuncture placed at a non-acupuncture point.^86^

A previous study^47^ inspired the development of the communication model, and the adopted positive communication style also is in line with a literature review regarding beneficial positive communication messages.^49^ To ensure the feasibility of the procedures for treatment and communication, these study procedures were tested prior to the present study in a non-cancer population.^51^ This involved, for example, ensuring successfully strengthened treatment expectations using the communication model, and credible blinding of patients using the telescoping sham needle. The lesson learned from that study was that, although the treatment and communication procedures were standardized, considerable differences were observed between intervention therapists.^51^ Data on the characteristics of the therapists thus need to be collected and analyzed in the GLMM. In the present study, we have limited the number of intervention therapists to a few therapists, and we are collecting detailed data on, for example, therapists’ experiences and treatment expectations.

Cancer care professionals’ views on whether communication strategies should be used to foster positive treatment expectations and improve health outcomes vary considerably.^87^ This study will therefore contribute valuable evidence on 2 fronts: the potential benefit of therapists’ communication styles and the efficacy of adding antiemetic acupuncture to standard care in reducing nausea and vomiting, enhancing QoL, and improving patients’ capacity for daily and physical activities. It will also provide insights into the possible psychological and biological mechanisms through which acupuncture affects nausea and vomiting. Implementation of the results in clinical practice will be facilitated by the close collaboration between researchers and clinicians as well as the fact that the study is being conducted in a clinical oncology setting, possibly creating a pathway between research findings and clinical practice.

To conclude, this trial will contribute to the understanding of how communication can strengthen treatment expectations and thereby alleviate chemotherapy-induced nausea and vomiting. If proven effective, the communication model aimed at enhancing positive treatment expectations in patients at risk of nausea and vomiting could be implemented in routine clinical care as part of side-effect management for patients with cancer.
